# CLEC11A improves insulin secretion and promotes cell proliferation in human beta-cells

**DOI:** 10.1530/JME-22-0066

**Published:** 2023-06-21

**Authors:** Ruifeng Shi, Jing Cen, Gunilla T Westermark, Sheng Zhao, Nils Welsh, Zilin Sun, Joey Lau

**Affiliations:** 1 Department of Endocrinology, First Affiliated Hospital of Anhui Medical University, Hefei, China; 2 Department of Endocrinology, Zhongda Hospital, Institute of Diabetes, School of Medicine, Southeast University, Nanjing, China; 3 Department of Medical Cell Biology, Uppsala University, Uppsala, Sweden; 4 Department of Biochemistry and Molecular Biology, School of Medicine, Southeast University, Nanjing, China

**Keywords:** CLEC11A, human islets, EndoC-βH1 cells, beta-cell function, proliferation

## Abstract

Beta-cell dysfunction is a hallmark of disease progression in patients with diabetes. Research has been focused on maintaining and restoring beta-cell function during diabetes development. The aims of this study were to explore the expression of C-type lectin domain containing 11A (CLEC11A), a secreted sulphated glycoprotein, in human islets and to evaluate the effects of CLEC11A on beta-cell function and proliferation *in vitro.* To test these hypotheses, human islets and human EndoC-βH1 cell line were used in this study. We identified that CLEC11A was expressed in beta-cells and alpha-cells in human islets but not in EndoC-βH1 cells, whereas the receptor of CLEC11A called integrin subunit alpha 11 was found in both human islets and EndoC-βH1 cells. Long-term treatment with exogenous recombinant human CLEC11A (rhCLEC11A) accentuated glucose-stimulated insulin secretion, insulin content, and proliferation from human islets and EndoC-βH1 cells, which was partially due to the accentuated expression levels of transcription factors *MAFA* and *PDX1*. However, the impaired beta-cell function and reduced mRNA expression of *INS* and *MAFA* in EndoC-βH1 cells that were caused by chronic palmitate exposure could only be partially improved by the introduction of rhCLEC11A. Based on these results, we conclude that rhCLEC11A promotes insulin secretion, insulin content, and proliferation in human beta-cells, which are associated with the accentuated expression levels of transcription factors *MAFA* and *PDX1*. CLEC11A, therefore, may provide a novel therapeutic target for maintaining beta-cell function in patients with diabetes.

## Introduction

Diabetes mellitus as a metabolic disease is becoming one of the major public health issues worldwide (Kahn *et al.* 2014). Obesity is believed to be the critical risk factor for developing type 2 diabetes, while type 1 diabetes is considered to be an autoimmune disease (DiMeglio *et al.* 2018, Schnurr *et al.* 2020). Despite the different causes of type 1 and type 2 diabetes (T2D), beta-cell dysfunction demonstrated by insufficient insulin release in response to hyperglycaemia has become a hallmark of disease progression in these patients (Greenbaum *et al.* 2009, Rosengren *et al.* 2012). Insulin, secreted from the beta-cells, is the only glucose-lowering hormone in the body and plays an important role in maintaining glucose homeostasis (Aronoff *et al.* 2004). The main goal in diabetic research has therefore been to focus on finding strategies and therapeutic targets to improve and restore beta-cell function in patients with diabetes.

C-type lectin domain containing 11A (CLEC11A), also called stem cell growth factor (SCGF) or C-type lectin superfamily member 3 or osteolectin1, is a secreted sulphated glycoprotein that is highly expressed in the bone marrows (Bannwarth *et al.* 1998, Mio *et al.* 1998). This protein was initially identified as a growth factor that promotes the proliferation and differentiation of hematopoietic progenitor cells *in vitro* (Hiraoka *et al.* 1997). During the past decades, it has been extensively studied in bone homeostasis, erythropoiesis, and haematopoiesis in animal models (Yue *et al.* 2016, Wang *et al.* 2020). In clinical research, the gene expression levels of *CLEC11A* have been related to the prognosis of adult acute myeloid leukaemia (Yin *et al.* 2021) and childhood acute lymphoblastic leukaemia (Silveira *et al.* 2013). Furthermore, integrin subunit alpha 11 (ITGA11) recently identified as a receptor for CLEC11A is strongly expressed in leptin receptor-positive skeletal stem cells and osteoblasts (Shen *et al.* 2019). CLEC11A is identified to be expressed in pancreatic islets from both mouse and human (Baron *et al.* 2016). In our previous study, RNA sequencing of mouse pancreatic islets revealed that the expression level of mouse *Clec11a* was downregulated in diet-induced obese mice compared with normal control mice. Moreover, CLEC11A was identified to play a regulatory role in proliferation and lipid metabolism in mouse islets (Shi *et al.* 2019). However, whether these results generated in mice would translate into human islets and how CLEC11A signals to human beta-cells have not been tested yet. Therefore, in this study, we aim to explore the expression and localization of CLEC11A in human islet insulin- and glucagon-producing cells and also to evaluate the effects of CLEC11A on beta-cell function and proliferation *in vitro.*

## Materials and methods

### Human islet and EndoC-βH1 cell culture

Isolated human islets from brain-dead, non-diabetic donors were generously provided from the Nordic Network for Clinical Islet Transplantation (Uppsala University Hospital, Uppsala, Sweden) or purchased from Prodo Laboratories, Inc. (Aliso Viejo, CA, USA). In total, islets from 15 donors (Supplementary Table 1, see section on supplementary materials given at the end of this article) were used in this study (age: 60.5 ± 2.1 years, male/female: 8/7; BMI: 26.3 ± 0.9 kg/m^2^, HbA1c: 37.6 ± 0.8 mmol/mol). Human islets were cultured in CMRL1066 medium (Gibco) supplemented with 10% fetal bovine serum (Gibco), 100 units/mL penicillin–streptomycin (Sigma Aldrich), and 1% glutamine (Sigma Aldrich) at 37°C in humidified air containing 5% CO_2_. Islets were used within 7 days after isolation. Ethical permission to use human islets has been obtained from the Regional Ethical Review Board in Uppsala, Sweden (Regionala etikprövningsnämnden, Uppsala, Sweden, date of approval 9 August 2017, project code nr 2017-283).

EndoC-βH1 cells were cultured in Geltrex^TM^ (Gibco)-coated plates in DMEM/Ham's F12 (Gibco) (50%/50%, vol/vol, 5.5 mM glucose) and supplemented as previously described (Krizhanovskii *et al.* 2017). This culture medium is hereafter referred to as basal culture media. Cells were cultured at 37°C in humidified air containing 5% CO_2_.

### Preparation of recombinant human CLEC11A protein and sodium palmitate

Recombinant human CLEC11A (rhCLEC11A) protein (R&D Systems) was dissolved in PBS (Gibco) containing 0.1% bovine serum albumin (BSA) (Sigma Aldrich) to a stock solution of 100 μg/mL. Different concentrations of rhCLEC11A were prepared by diluting the stock solution in the basal culture medium.

Sodium palmitate (Sigma Aldrich) was prepared by dissolving in 50% ethanol to make a stock solution with a concentration of 100 mM as previously described (Cen *et al.* 2018). The stock solution was then diluted in basal culture medium and allowed to form a complex with fatty acid-free BSA (Sigma Aldrich) at 37°C for at least 30 min. The final palmitate concentration (1.5 mM with 2% BSA) prepared for EndoC-βH1 cell treatment results in a fatty acid/BSA molar ratio of 4.9:1, and this ratio has been studied in EndoC-βH1 cells *in vitro* (Krizhanovskii *et al.* 2017).

### Immunofluorescence

For the co-localization experiment of CLEC11A with insulin or CLEC11A with glucagon, immunolabelling was performed on partially digested human islets. Briefly, islets were trypsin treated for 5–8 min, and smaller cell aggregates were cytospinned (175 ***g*** for 3 min) onto polylysine pre-coated slides, followed by fixation in 4% PFA for 5 min. After washing with PBS three times, the cells were permeabilized in 0.1% Triton X-100 for 10 min, followed by blocking in 2% fetal calf serum (FCS) for 60 min and then incubated for 60 min at 37°C with the primary antibodies. Following washing with PBS twice and then Dako washing buffer (Agilent) once, all slides were incubated with diluted secondary antibodies for 1 h at room temperature. Cells were then washed four times with PBS and mounted with VECTASHIELD Hard Set mounting medium with DAPI (Vector Laboratories, Newark, NJ, USA). Images were acquired using confocal microscopy (Nikon).

Primary antibodies were mouse anti-human SCGF/CLEC11A (1:300) (R&D Systems), guinea pig anti-human insulin (1:300) (Fitzgerald, Acton, MA, USA), and goat anti-human glucagon (1:300) (Abbexa, Cambridge, UK). Secondary antibodies were Alexa Fluor 594 donkey anti-mouse (1:300) (Jackson ImmunoResearch), Alexa Fluor 488 donkey anti-guinea pig (1:300) (Jackson ImmunoResearch), and Alexa Fluor 488 rabbit anti-goat (20 μg/mL) (Invitrogen).

### Cell proliferation

The proliferation rate of beta-cells in human islets was analysed using the Click-iT EdU Cell Proliferation Kit for Imaging (Invitrogen) according to the manufacturer’s instruction. EdU is used to assay DNA synthesis in cell culture and detect cells undergoing DNA synthesis. This method has been applied on human islets (Maachi *et al.* 2021). Briefly, human islets (20–30 islets/group) were treated with different concentrations of rhCLEC11A (0 ng/mL, 10 ng/mL, 100 ng/mL, or 1000 ng/mL) for 2 days and rhCLEC11A (0 ng/mL, 10 ng/mL, or 100 ng/mL) for 7 days. EdU (10 µM) was added during the rhCLEC11A treatment period. EdU-positive cells were labelled with Alexa Fluor 488 dye. The proliferation rate of EndoC-βH1 cells was assessed by the nuclear protein Ki67 staining, which is widely used as a proliferation marker. Immunodetection of Ki67 labelling in EndoC-βH1 cells was carried out on cells seeded on coverslips and treated with rhCLEC11A (10 ng/mL) for 2 days. Immunostaining of insulin and Ki67 was performed according to the protocol described earlier (Espes *et al.* 2015). Images were acquired by confocal microscopy (Zeiss LSM780) and analysed using Image J software. In human islets, the proportion of EdU-positive cells of insulin-positive beta-cells was determined. In EndoC-βH1 cells, the percentage of Ki67-positive beta-cells was analysed.

The antibodies used for human islets were primary guinea pig anti-human insulin (1:300) (Fitzgerald) and secondary Alexa Fluor 647 donkey anti-guinea pig (1:300) (Jackson ImmunoResearch). The antibodies used for EndoC-βH1 cells were primary guinea pig anti-human insulin (1:300) (Fitzgerald) and primary rabbit anti-Ki67 (1:500) (Abcam), followed by corresponding secondary antibodies of Alexa Fluor 488 donkey anti-guinea pig (1:300) (Jackson ImmunoResearch) and Cy3 donkey anti-rabbit (1:300) (Jackson ImmunoResearch), respectively.

### Glucose-stimulated insulin secretion from human islet and EndoC-βH1 cell

Krebs Ringer bicarbonate buffer (KRBH) buffer supplemented with 0.1% BSA and adjusted to pH 7.4 as previously described (Cen *et al.* 2018) was used to study the static incubation of glucose-stimulated insulin secretion (GSIS) at 37°C in both human islets and EndoC-βH1 cells. Human islets in triplicates (ten islets per replicate) from different treatments were handpicked and pre-incubated in KRBH buffer with 1.67 mM glucose for 90 min, followed by incubation at low glucose concentration (1.67 mM glucose) for 60 min and then with high glucose concentration (16.7 mM glucose) for 60 min. Supernatants from low and high glucose concentrations were collected for insulin secretion measurement. A total of 30 islets from the same treatment group were harvested together and homogenized by sonicating in 200 µL of redistilled H_2_O, and an aliquot was used for DNA content measurement. From the sonicated sample, 50 µL solution were mixed with 125 µL of 95% acid ethanol and then stored at –20 °C until measurement of insulin content. Insulin concentration was measured by Insulin ELISA Assay Kit (Mercodia, Uppsala, Sweden). DNA content was measured by the PicoGreen dsDNA quantitation assay (Invitrogen).

EndoC-βH1 cells were seeded in 48-well plates and cultured to reach 90% confluency before GSIS experiments. After different treatments, cells were pre-incubated in KRBH containing 0.1% BSA and 2 mM glucose for 60 min. Thereafter, cells were incubated in 2 mM glucose in KRBH for 30 min followed by incubation in 20 mM glucose in KRBH at 37°C for 30 min. Supernatants were collected for the analysis of the amount of secreted insulin. For the experiment exploring the acute effect of rhCLEC11A in EndoC-βH1 cells, the static GSIS protocol was modified by the addition of different concentrations of rhCLEC11A during the 20 mM glucose incubation. After glucose exposure, cells were washed with Dulbecco's phosphate-buffered saline (DPBS) (Gibco) and further lysed in DPBS containing 1% Triton X-100 (Sigma Aldrich) at 4°C overnight. Lysates were used to measure the insulin content and total protein concentration. Insulin concentration was measured by Insulin ELISA Assay Kit (Mercodia). Protein content was measured by DC protein assay (Bio-Rad).

### WST-1 assay

EndoC-βH1 cells (1 × 10^3^) were seeded and cultured in 96-well plates with 100 µL/well basal culture media (5.5 mM glucose) for 1 day, followed by treatment with different concentrations of rhCLEC11A (10 ng/mL, 100 ng/mL, and 1000 ng/mL, respectively) for 48 h. The cell proliferation was assessed using water-soluble tetrazolium salt (WST-1) reagent (Abcam) by directly measuring absorbance at 440 nm after WST-1 culture for 2 h. The absorbance is proportional to the cell number.

### Quantitative real-time PCR

Total RNA of human islets and cells was extracted using RNeasy Plus Micro Kit (Qiagen), and cDNA was synthesized using 1 µg RNA of each sample and reverse transcribed using SuperScript First-Strand Synthesis SuperMix (Invitrogen) according to the manufacturer's protocol. Quantitative RT-PCR (RT-qPCR) was performed using PowerUp SYBR Green Master Mix (Applied Biosystems) on QuantStudio 5 Real-Time PCR Systems (Applied Biosystems). *GAPDH* and *ACTB* were used as endogenous controls. Relative gene expression levels of *CLEC11A* and *ITGA11* in human islets and EndoC-βH1 cells were normalized by subtracting the geometric average *C_t_* value of these two endogenous controls (Vandesompele *et al.* 2002) and further calculated with the 2^–△Ct^ method. Relative gene expressions in rhCLEC11 treatment experiments in human islets and EndoC-βH1 cells were normalized by subtracting the *C_t_*value of *ACTB* and *GAPDH*, respectively, from the *C_t_* value for the gene studied. Relative mRNA expression of the gene studied was calculated with the 2^–△△Ct^ method. Primers were synthesized by Integrated DNA Technologies, and the sequences are listed in Supplementary Table 1.

### Western blot

Total protein was collected from human islets and EndoC-βH1 cells using Pierce RIPA Buffer (Thermo Fisher) and Protease Inhibitor Cocktail (Sigma Aldrich). The protein was measured using Bio-Rad DC Protein Assay (Bio-Rad). An equal amount of protein was separated by Mini-PROTEAN TGX Stain-Free Gels (Bio-Rad) and transferred to a PVDF membrane. After blocking with 5% non-fat milk, the membrane was incubated with diluted primary antibodies overnight at 4°C. Primary antibodies used for western blot were as follows: mouse anti-human SCGF/CLEC11A (1:500) (R&D Systems), rabbit anti-human ITGA11 (1:500) (LSBio, Shirley, MA, USA), rabbit anti-human PDX1 (1:3000) (Abcam), and rabbit anti-beta-actin (1:1000) (Cell Signaling Technology). After washing and incubation with a horseradish peroxidase (HRP)-labelled secondary goat anti-rabbit antibody (1:1000) (Thermo Fisher) for 60 min, reactivities were detected by an HRP chemiluminescent substrate kit (Bio-Rad). Positive signals were quantified using Image J software. For immunofluorescence western blot, the immunodetection was performed using IRDye 800CW- (green) and IRDye 680 RD (red)-labelled secondary antibodies diluted 1:15 000, respectively (LI-COR, Lincoln, NE, USA), and fluorescence was recorded and analysed in the Odyssey FC instrument (LI-COR).

### Exploring RNA-sequencing database of human islets

Islet Gene View (IGW) platform (https://mae.crc.med.lu.se/IsletGeneView/) was scrutinized for data on the expression of *CLEC11A* gene in human islets. IGW is a web tool based on the RNA-sequencing and genome-wide genotyping in human islets from 188 donors (155 non-T2D and 33 T2D). The IGW web application produces a robust overview of data and can produce informative graphs for a particular gene of interest (Asplund *et al.* 2022).

### Data analysis

Data were analysed using GraphPad Prism9 (GraphPad software). The results generated from biological replicates were expressed as mean of independently repeated experiments ± s.e.m.. For comparison of two groups, the Student’s *t*-test was used and for comparison of several groups one-way ANOVA or two-way ANOVA followed by Holm–Sidak multiple comparison test was used. A *P-*value < 0.05 was considered statistically significant.

## Results

### Expression of CLEC11A and ITGA11 in human islets and EndoC-βH1 cells

**Figure 1:**
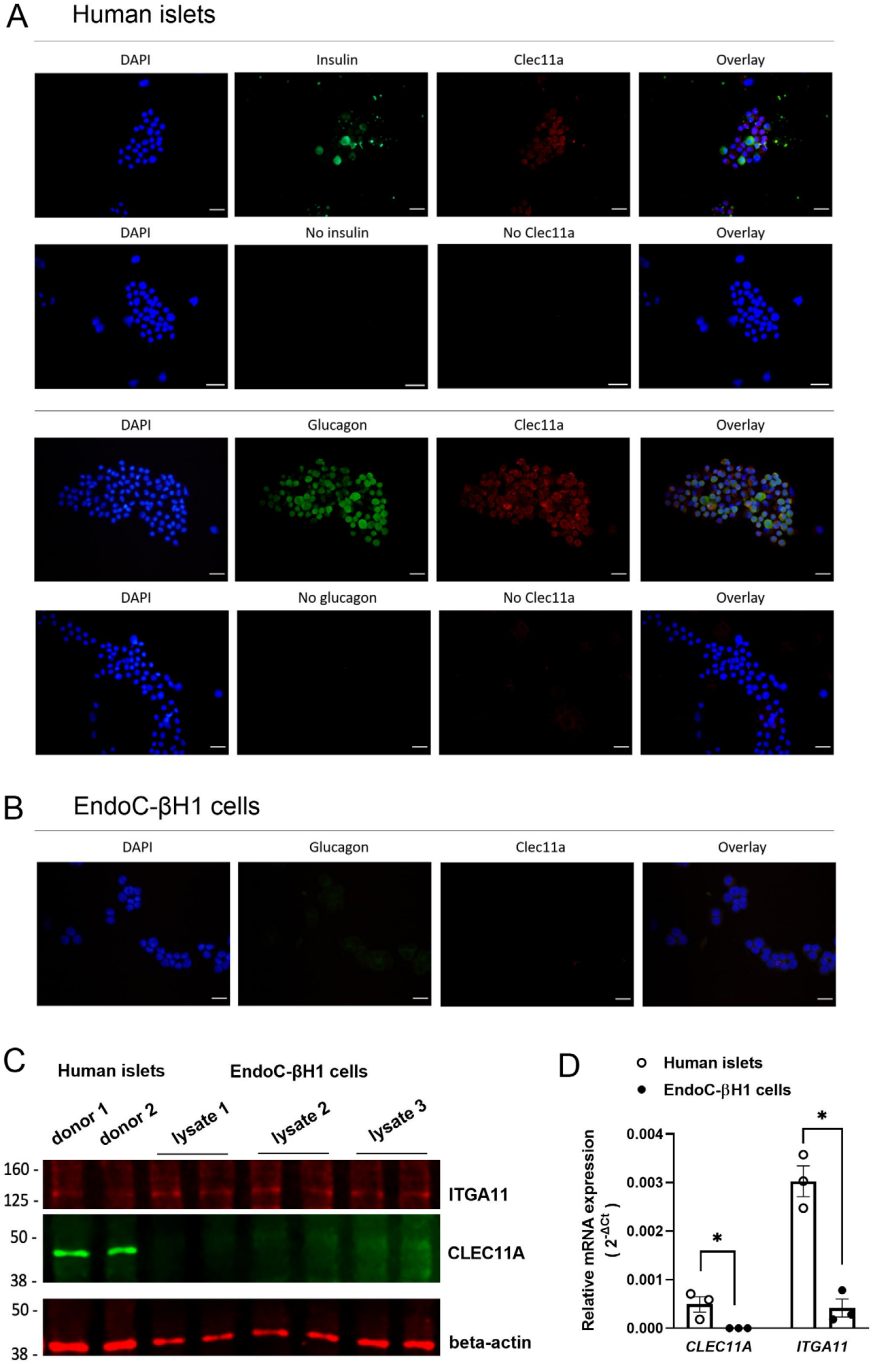
Expression of CLEC11A and its receptor ITGA11 in human islets and EndoC-βH1 cells. (A) Representative immunofluorescence staining of partially disrupted human islets revealed the expression and co-localization of CLEC11A (red) with insulin-secreting beta-cells (green) or with glucagon-secreting alpha-cells (green), respectively. Nuclei were stained with DAPI (blue). The negative control groups of disrupted human islets without primary antibodies were also shown. Scale bars, 50 μm. (B) Representative immunofluorescence staining of EndoC-βH1 cells. The staining of CLEC11A (red) and glucagon (green) was not detected in EndoC-βH1 cells. Scale bars, 50 μm. (C) Western blot and (D) RT-qPCR confirmed the expression of CLEC11A in human islets but not in EndoC-βH1 cells, whereas its receptor ITGA11 was found to be expressed in both human islets and EndoC-βH1 cells. The relative mRNA expression levels of *CLEC11A* and *ITGA11* were first normalized by using the geometric average *C_t_* value of two endogenous controls *GAPDH* and *ACTB*, respectively, and further calculated with the 2^–△Ct^ method. Results are expressed as mean ± s.e.m. of *n* = 3 donors. *Indicates *P* < 0.05.

The expression of CLEC11A was investigated in partially dispersed human islets, and the results showed that endogenous CLEC11A was expressed in human islets and localized in both insulin-secreting beta-cells and glucagon-secreting alpha-cells (Fig. 1A), whereas in EndoC-βH1 cells, endogenous CLEC11A could not be detected by immunofluorescence staining (Fig. 1B). This difference in expression pattern was further confirmed by western blot (Fig. 1C) and RT-qPCR (Fig. 1D), where CLEC11A was shown to be expressed in human islets but not in EndoC-βH1 cells. The expression of ITGA11 as the receptor of CLEC11A was also explored in this study. ITGA11 was expressed in both human islets and EndoC-βH1 cell at the protein level and the mRNA level, as assessed by western blot (Fig. 1C) and RT-qPCR (Fig. 1D), respectively. However, the mRNA expression level of* ITGA11* detected in EndoC-βH1 cells was low and estimated to be 15% of the expression level of *ITGA11* determined in human islets (Fig. 1D).

In the IGW platform (Asplund *et al.* 2022), the *CLEC11A* gene was detected in human islets, as shown by histogram of gene FPKMs (fragments per kilobase of transcript per million mapped reads) (Supplementary Fig. 1A). The single cell sequencing data of human islets (Segerstolpe *et al.* 2016) also included in the IGW platform showed that different types of cells in human islets express *CLEC11A* but at different levels. Among them, the expression level of *CLEC11A* in alpha-cells is higher than that in beta-cells (Supplementary Fig. 1B). Changes in *CLEC11A* gene expression in human islets from T2D and control donors were also investigated. The results showed that there was no significant difference in the expression level of *CLEC11A* in non-T2D donors and T2D donors of human islets (Supplementary Fig. 1C); however, *CLEC11A* and *INS* gene expression was positively correlated (Supplementary Fig. 1D). Thus, these data further provide compelling evidence for us to explore the function of CLEC11A on human beta-cells.

### Exogenous rhCLEC11A accentuated GSIS and insulin content in human islets and EndoC-βH1 cells

**Figure 2:**
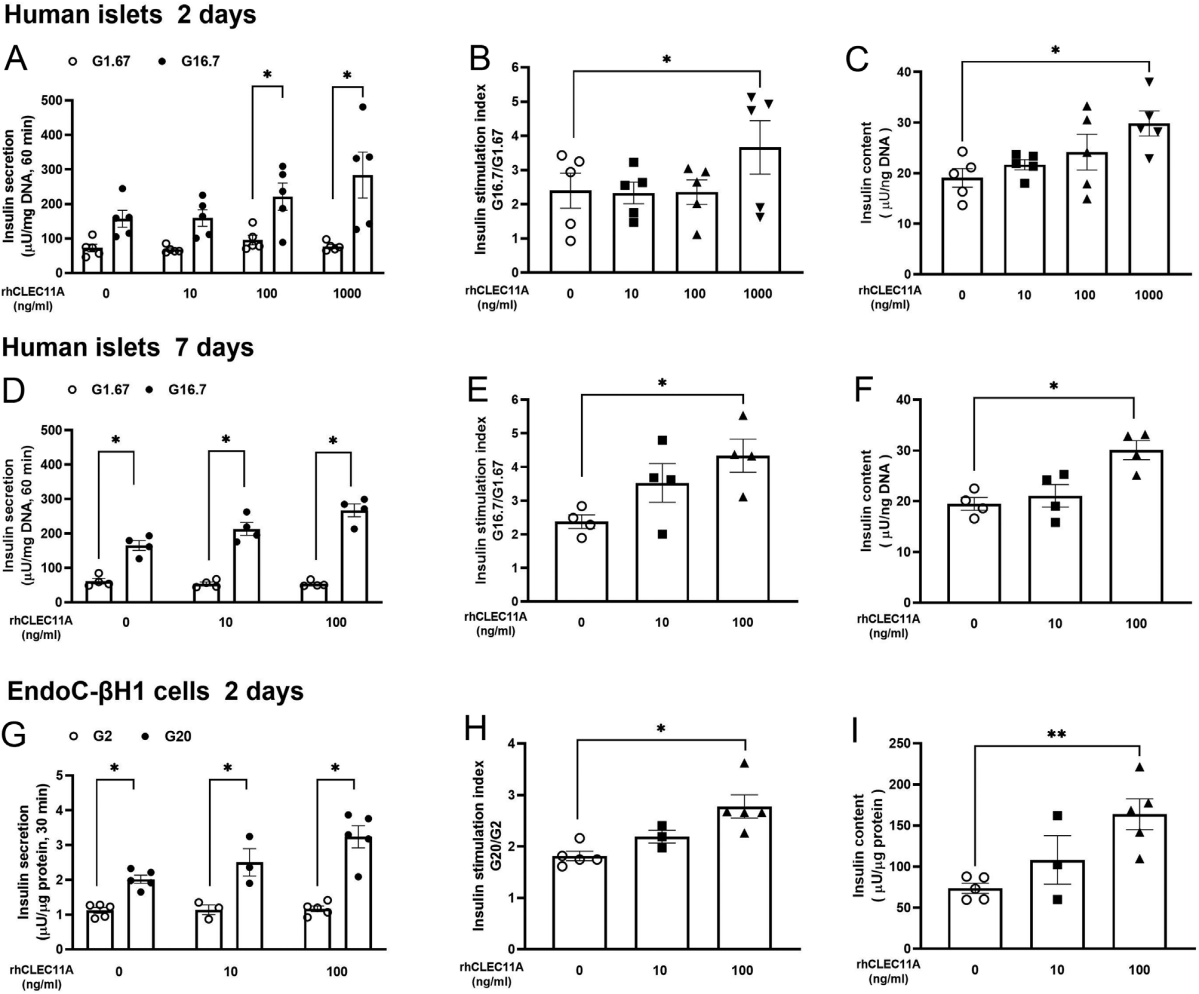
Effects of rhCLEC11A on GSIS and insulin content from human islets and EndoC-βH1 cells. (A) Human islets (10 islets/group, triplicates) were cultured in the absence or presence of different concentrations of rhCLEC11A (10, 100, and 1000 ng/mL, respectively) for 2 days. Static GSIS was performed on islets with 1.67 mM glucose (G1.67) and 16.7 mM glucose (G16.7) for 60 min, and secreted insulin was normalized to total DNA content. (B) Insulin stimulation index at 2-day culture was calculated. (C) After GSIS, human islets were lysed, and insulin content was normalized to the total DNA content. (D) Human islets were cultured in the absence or presence of different concentrations of rhCLEC11A (10 and 100 ng/mL, respectively) for 7 days. Insulin secreted from static GSIS was normalized to total DNA and (E) insulin stimulation index at 7-day culture was calculated. (F) Total insulin content was normalized to total DNA content. (G) EndoC-βH1 cells were cultured in the absence or presence of different concentrations of rhCLEC11A (10 and 100 ng/mL, respectively) for 2 days. After culture, static GSIS was performed on cells with 2 mM glucose (G2) and 20 mM glucose (G20) for 30 min and (H) insulin stimulation index was calculated. (I) EndoC-βH1 cells were lysed after GSIS, and insulin content was measured and normalized to total protein. Results are expressed as means ± s.e.m. of *n* = 4–5 donors from human islets and means ± s.e.m. of *n* = 3–5 independent experiments from EndoC-βH1 cells. *Indicates *P* < 0.05.

The dose-dependent effects of exogenous rhCLEC11A on beta-cell function were explored by static GSIS and insulin content from human islets and EndoC-βH1 cells, respectively. The insulin stimulation index was calculated from the amount of insulin secreted at 16.7 mM glucose incubation divided by the amount of insulin secreted at 1.67 mM glucose incubation from human islets treated with different concentrations of rhCLEC11A. Compared with the control islets, incubation with 1000 ng/mL of rhCLEC11A for 2 days significantly increased the islet insulin stimulation index (Fig. 2A and B).

Insulin content measured from the same islets showed that 1000 ng/mL of rhCLEC11A increased the islet content to approximately 1.5-fold of the control islets (Fig. 2C). We also explored the long-term effects of rhCLEC11A on human islet beta-cell function by extending the incubation time to 7 days, and similar results were obtained. After 7 days of culture, compared with the control human islets, 100 ng/mL of rhCLEC11A almost doubled the secretion of insulin (Fig. 2D and E) and significantly increased the insulin content by around 50% (Fig. 2F).

In order to dissect the contribution of beta-cells in rhCLEC11A-induced insulin secretion and insulin content observed in human islets, EndoC-βH1 cells were thus used to study the effects of rhCLEC11A on beta-cells alone. In line with the observations from human islets, EndoC-βH1 cells treated with different concentrations of rhCLEC11A for 2 days showed an increase in insulin secretion (Fig. 2G). When compared with the control group, the insulin stimulation index was not changed in the groups incubated with 10 ng/mL of rhCLEC11A but increased by about 40% in the group exposed to 100 ng/mL of rhCLEC11A (Fig. 2H). Insulin content in rhCLEC11A-treated EndoC-βH1 cells showed a similar pattern as determined in 7-day cultured human islets. After 2-day culture, insulin content was doubled in cells exposed to 100 ng/mL of rhCLEC11A compared with the control group (Fig. 2I). To test if exogenous rhCLEC11A can directly affect insulin secretion from beta-cells, different concentrations of CLEC11A (0, 10, 100, and 1000 ng/mL) were acutely administrated when cells were exposed to 20 mM glucose concentration during GSIS. The results showed that rhCLEC11A had no direct effect on GSIS in EndoC-βH1 cells regardless of the rhCLEC11A concentration (Supplementary Fig. 2A and B).

### Exogenous rhCLEC11A promotes beta-cell proliferation in human islets and EndoC-βH1 cells

**Figure 3:**
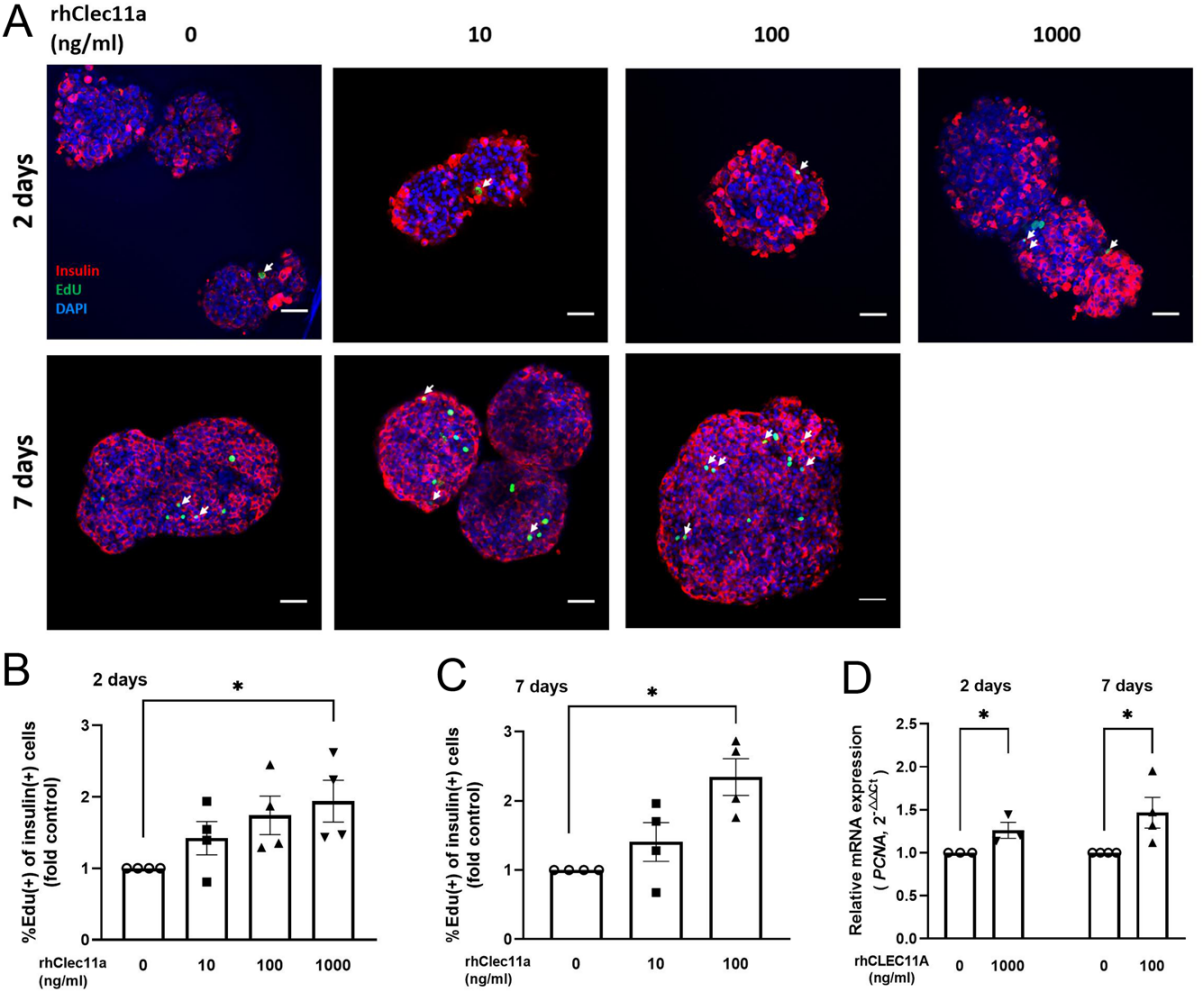
rhCLEC11A promotes beta-cell proliferation in human islets. Human islets (20–30 islets/treatment) were cultured in the absence or presence of different concentrations of rhCLEC11A indicated in the figures for 2 days and 7 days, respectively. (A) Representative image of immunofluorescence staining for EdU and insulin in human islets. EdU (green), insulin (red), and nuclei (blue) are shown. EdU-positive insulin-staining cells are indicated with arrows (white). Scale bars, 50 μm. The percentage of EdU-positive cells in insulin-staining beta-cells was calculated from the immunofluorescent images and expressed as fold control for (B) 2-day culture and (C) 7-day culture. (D) After 2-day and 7-day culture, the relative mRNA expression level of the *PCNA* gene from human islets was measured by RT-qPCR, normalized to endogenous control *ACTB* and calculated with the 2^–△△Ct^ method. Results are means ± s.e.m. of *n* = 3–4 donors from human islets. *Indicates *P* < 0.05.

CLEC11A has been linked to hematopoietic progenitor cell proliferation (Hiraoka *et al.* 1997), and our previous study in mouse islets and murine MIN6 beta-cells showed an increased cell proliferation rate stimulated by the administration of rhCLEC11A (Shi *et al.* 2019). Cell proliferation in human islet beta-cells was studied by combining the EdU assay and insulin staining on human islets cultured for 2 days and 7 days, respectively, in the absence and presence of rhCLEC11A (Fig. 3A). Compared with the control islets, the culture of islets in 10 µM EdU for 2 days and 7 days did not induce cell death (Supplementary Fig. 3). The calculated proliferation rate of human islet beta-cells showed a dose-dependent increase. Culture in 1000 ng/mL of rhCLEC11A for 2 days almost doubled the percentage of EdU-positive insulin-producing beta-cells compared with untreated control islets (Fig. 3B). Accordingly, a similar increase was obtained by 100 ng/mL of rhCLEC11A-treated human islets after 7 days of culture (Fig. 3C). The increased beta-cell proliferation rate was further confirmed by measuring the gene expression of proliferating cell nuclear antigen (PCNA), which plays an essential role in nucleic acid metabolism as a component of the replication and repair machinery (Kelman 1997). The mRNA expression level of *PCNA* was measured by RT-qPCR. The *PCNA* gene expression was increased by almost 20% in human islets cultured in 1000 ng/mL of rhCLEC11A for 2 days (Fig. 3B) and was increased by approximately 50% in islets treated with 100 ng/mL of rhCLEC11A for 7 days (Fig. 3C), compared with the respective control group.

**Figure 4:**
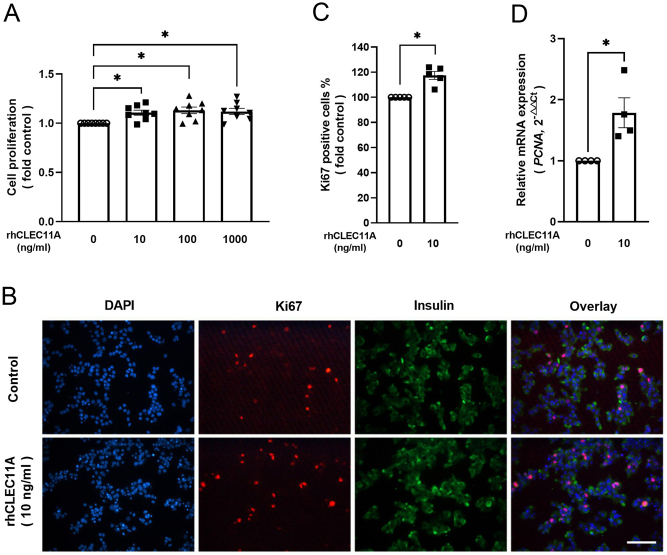
rhCLEC11A promotes the proliferation of EndoC-βH1 cells. EndoC-βH1 cells were cultured in the absence or presence of different concentrations (10, 100, or 1000 ng/mL, respectively) of rhCLEC11A for 2 days. (A) Proliferation was measured using a WST assay. (B) Representative image of immunofluorescence staining for Ki67 and insulin from EndoC-βH1 cells treated with or without rhCLEC11A (10 ng/mL) for 2 days. Ki67 (red), insulin (green), and nuclei (blue) are shown. Scale bars, 50 μm. (C) Ki67-positive cells were further calculated from immunofluorescence images. (D) After culture, the relative mRNA expression level of the *PCNA* gene was measured by RT-qPCR, normalized to endogenous control *GAPDH* and calculated with the 2^–△△Ct^ method. Results are means ± s.e.m. of *n* = 4–8 independent experiments from EndoC-βH1 cells. * indicates *P* < 0.05.

The proliferation rate of EndoC-βH1 cells assessed by WST assay was increased by 10% in culture with rhCLEC11A for 2 days compared with the control cells regardless of the rhCLEC11A concentrations tested (Fig. 4A). This result was in line with the percentage of the Ki67-positive cells (Fig. 4C), which was determined after immunostaining in EndoC-βH1 cells (Fig. 4B). Compared with the control cells, rhCLEC11A (10 ng/mL) accentuated the mRNA expression level of *PCNA* by around 80% (Fig. 4D).

### Exogenous rhCLEC11A increases the expression levels of transcription regulators *MAFA* and *PDX-1* in human islets and EndoC-βH1 cells

**Figure 5:**
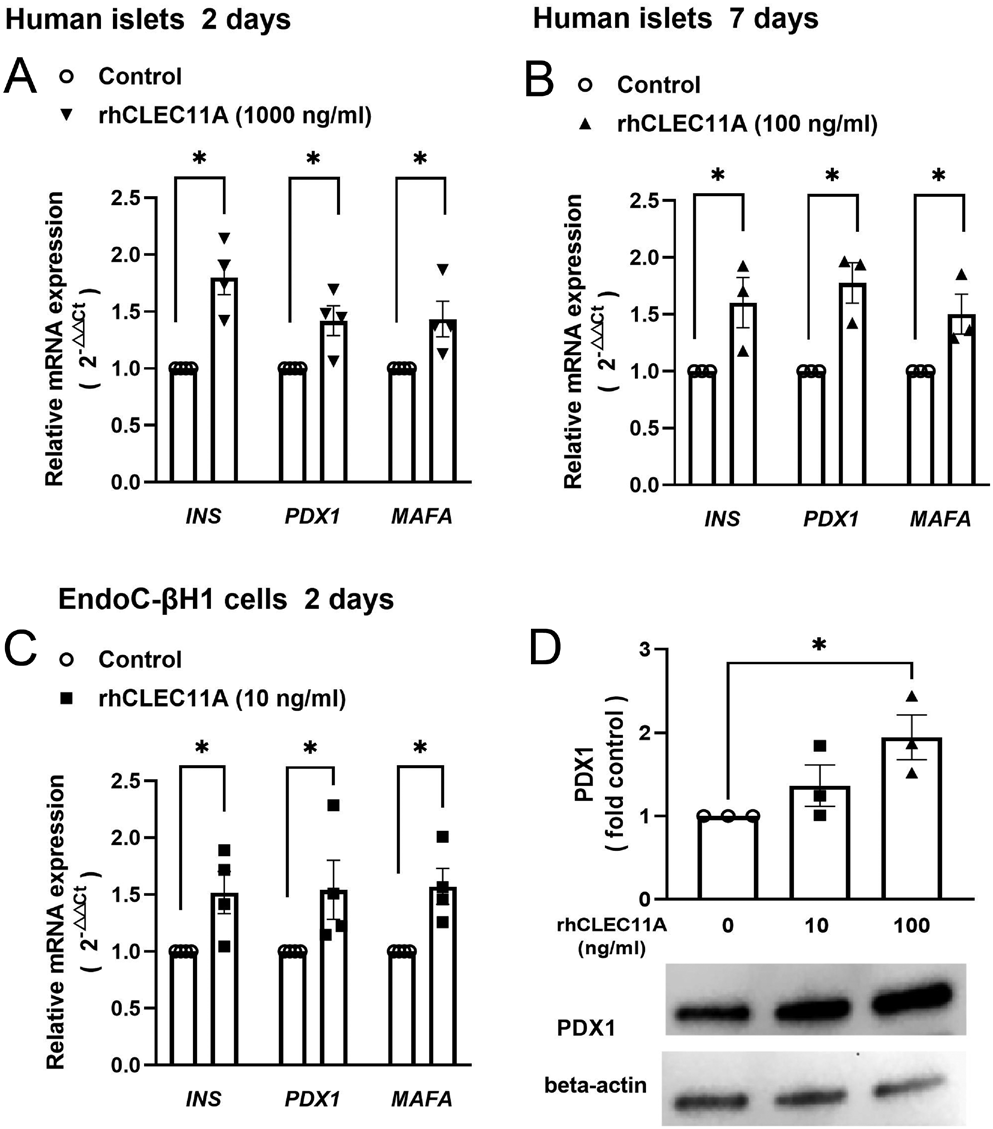
rhCLEC11A induces the expression of transcription factors in human islets and EndoC-βH1 cells. The relative mRNA expression levels of genes related to insulin (*INS*) and transcription factors (*PDX1 and MAFA*) were measured by RT-qPCR, normalized to endogenous control *ACTB* (for human islets) and *GAPDH* (for EndoC-βH1 cells), and calculated with the 2^–△△Ct^ method, respectively. Respective gene expression in human islets with (A) 2-day culture in the absence or presence of 1000 ng/mL of rhCLEC11A and (B) 7-day culture in the absence or presence of 100 ng/mL of rhCLEC11A. (C) Respective gene expression in EndoC-βH1 cells cultured in the absence or presence of 10 ng/mL of rhCLEC11A for 2 days. (D) EndoC-βH1 cells were cultured in the absence or presence of different concentrations of rhCLEC11A (10 and 100 ng/mL, respectively) for 2 days. The expression level of PDX1 was confirmed by western blot, normalized to beta-actin and expressed as fold control. Results are expressed as means ± s.e.m. of *n* = 3–4 donors from human islets and means ± s.e.m. of *n* = 3–4 independent experiments from EndoC-βH1 cells. *indicates *P* < 0.05.

To explore the underlying mechanisms for elevated insulin secretion and proliferation in human islet beta-cells and EndoC-βH1 cells under long-term exposure to rhCLEC11A, V-maf musculoaponeurotic fibrosarcoma oncogene homolog A (MAFA) and pancreatic and duodenal homeobox 1 (PDX1) were chosen and further investigated. MAFA and PDX1 are two well-known transcription factors that have been reported not only to be implicated in beta-cell development and maturation (Zhu *et al.* 2017) but also to play a role in insulin biosynthesis and secretion in murine islets and beta-cells (Hagman *et al.* 2005, Zhu *et al.* 2017). In this study, we first evaluated the expression levels of these two genes in human islets from both 2-day and 7-day culture and explored if they were affected by long-term treatment with exogenous rhCLEC11A. Compared with the control islets, mRNA expression levels of both *MAFA* and *PDX1* were increased by ~40% in human islets incubated with 1000 ng/mL of rhCLEC11A for 2 days (Fig. 5A), which was in line with the *INS* mRNA expression level (Fig. 5A) and insulin content (Fig. 2C). Accordingly, human islets cultured with 100 ng/mL of CLEC11A for 7 days (Fig. 5B) and EndoC-βH1 cells treated with 10 ng/mL of CLEC11A for 2 days (Fig. 5C) revealed a similar expression pattern for genes, i.e. *INS*, *PDX1*, and *MAFA*. Furthermore, the protein expression level of PDX1 in EndoC-βH1 cells was induced by long-term rhCLEC11A treatment (Fig. 4D). Therefore, increased expression of *MAFA* and *PDX1* caused by long-term rhCLEC11A treatment may contribute to the increased GSIS and insulin content in both human islets and in EndoC-βH1 cells.

### Exogenous rhCLEC11A partially improves beta-cell function from chronic palmitate-exposed EndoC-βH1 cells

**Figure 6:**
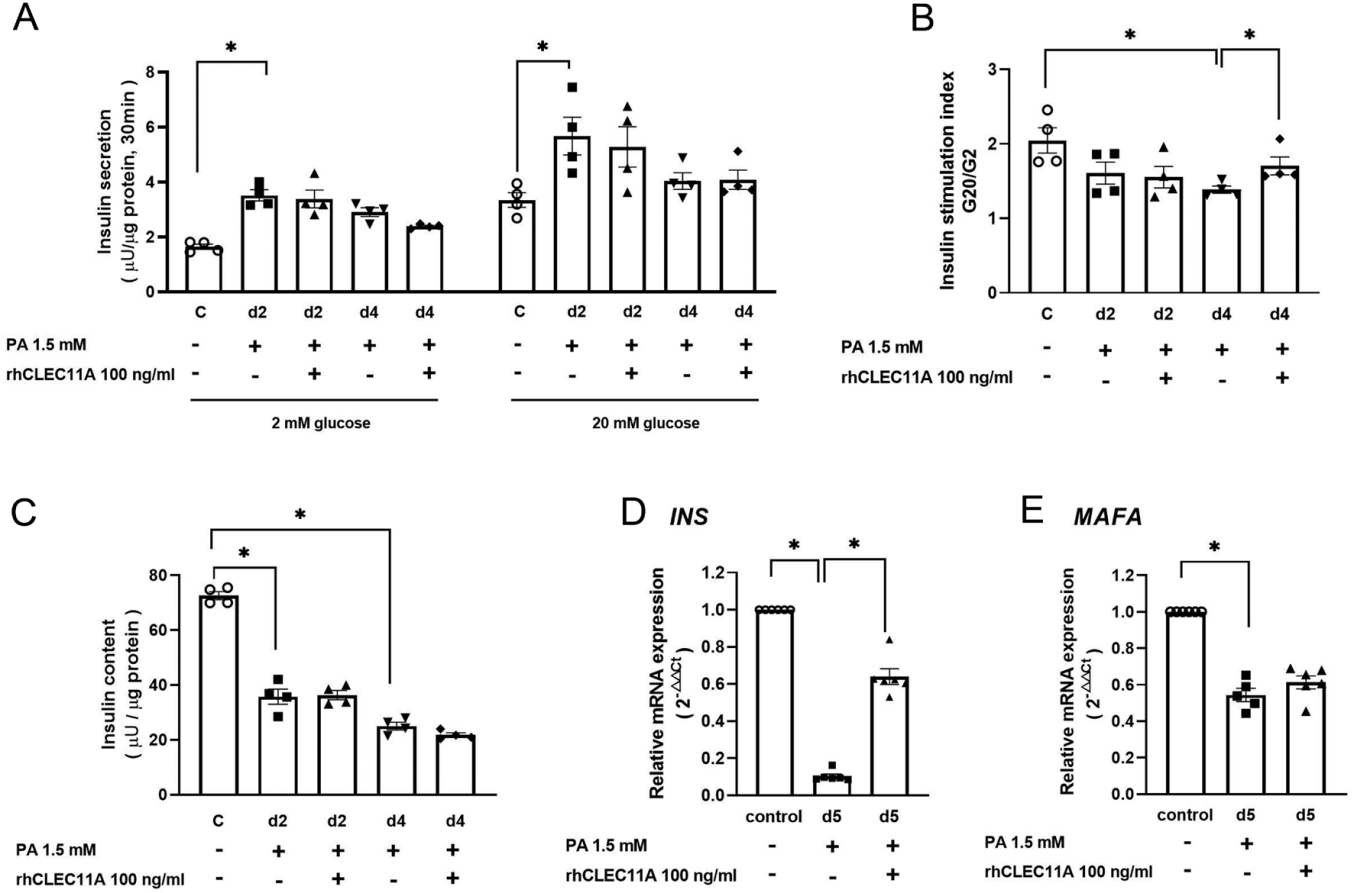
Effects of CLEC11A on EndoC-βH1 cells under chronic palmitate exposure. EndoC-βH1 cells were cultured in the absence or presence of 1.5 mM palmitate (PA) with or without 100 ng/mL of CLEC11A for different time periods. After culture, (A) static GSIS was performed with 2 mM glucose (G2) and 20 mM glucose (G20) for 30 min and (B) insulin stimulation index was calculated. (C) EndoC-βH1cells were lysed after GSIS, and insulin content was measured and normalized to total protein. After culture, the relative mRNA expression levels of (D) *INS* and (E) *MAFA* were measured by RT-qPCR, normalized to endogenous control *GAPDH* and calculated with the 2^–△△Ct^ method, respectively. Results are expressed as means ± s.e.m. of *n* = 4–5 independent experiments from EndoC-βH1 cells. * indicates *P* < 0.05.

Next, the potential protective effects of rhCLEC11A were explored in EndoC-βH1 cells exposed to the high levels of palmitate, which is established as an *in vitro* model for studies of beta-cell lipotoxicity (Krizhanovskii *et al.* 2017). Analysis of time-dependent effects from the high level of palmitate (1.5 mM with 2% BSA) incubation on EndoC-βH1 cells was performed to identify the time point when lipotoxicity was initiated. As we expected, high levels of palmitate caused a time-dependent decrease in the mRNA expression of *INS*, *MAFA*, and *PCNA*, respectively (Supplementary Fig. 4A, B and C). Based on these results, the impact of rhCLEC11A on insulin secretion and intracellular insulin content from cells exposed to palmitate for 2 days and 4 days was chosen and further studied. Palmitate alone accentuated insulin secretion at both low (2 mM) and high (20 mM) glucose levels after 2-day culture (Fig. 6A), whereas insulin stimulation index showed a time-dependent decrease from palmitate-treated cells. The introduction of rhCLEC11A did not affect insulin stimulation index in cells after a 2-day culture but improved insulin stimulation index by approximately 25% after a 4-day culture, when compared with the palmitate-cultured cells at the respective time points (Fig. 6B). In line with the insulin stimulation index pattern from palmitate-treated cells, insulin content showed a similar pattern. However, the introduction of rhCLEC11A could not improve palmitate-decreased insulin content (Fig. 5C). The effects of rhCLEC11A on gene expression in palmitate-treated cells were investigated. Palmitate alone reduced the mRNA expression levels of *INS* and *MAFA* after a 5-day culture, respectively. The administration of rhCLEC11A significantly improved *INS* mRNA expression but had no impact on *MAFA* expression from palmitate-exposed cells (Fig. 6D and E).

## Discussion

In this study, we have detected the expression and co-localization of CLEC11A in insulin-secreting beta-cells and glucagon-secreting alpha-cells in human islets. CLEC11A accentuated insulin secretion and promoted cell proliferation in human beta-cells in both human islets and human EndoC-βH1 cell line, which is probably via an osteolectin receptor ITGA11 followed by the induced expression of transcription regulators MAFA and PDX1. However, CLEC11A has weak effects in protecting EndoC-βH1 cells against chronic palmitate-induced cell dysfunction.

Based on our previous findings that CLEC11A plays a regulatory role in proliferation and lipid metabolism in mouse islets and in a mouse beta-cell line (Shi *et al.* 2019), this study was performed to explore the effects of CLEC11A in human beta-cell function and to investigate the potential underlying mechanisms. Therefore, as a first step, the expression and localization of CLEC11A were investigated in both human islets and EndoC-βH1 cells. We found that CLEC11A and ITGA11 are expressed in human islets, which were in line with the reported human islet single-cell sequencing results (Segerstolpe *et al.* 2016, Asplund *et al.* 2022). The expression level of *CLEC11A* is described to be higher in alpha-cells than that in beta-cells in the available online IGW web tool (Asplund *et al.* 2022). But surprisingly, EndoC-βH1 cells express ITGA11 but not CLEC11A, which provided us with a human beta-cell model where we could study the effects of rhCLEC11A without the interference of endogenous CLEC11A. The EndoC-βH1 cell line is a human beta-cell line and has been widely used to study the beta-cell function *in vitro* (Andersson *et al.* 2015, Krizhanovskii *et al.* 2017). Therefore, both human islets and EndoC-βH1 cells were used to further investigate the effects and underlying mechanisms of CLEC11A in human beta-cells.

In order to find the proper concentration of rhCLEC11A that could be applied in this *in vitro* study, we tested the effects of rhCLEC11A on beta-cell function in both human islets and EndoC-βH1 cells in a dose-dependent manner. The serum CLEC11A levels in healthy individuals are between 10 and 80 ng/mL, and this correlates with the haemoglobin levels (Ito *et al.* 2003, Ouma *et al.* 2010). The rhCLEC11A concentration of 10–1000 ng/mL was thus chosen and tested in different experiments. Long-term culture of rhCLEC11A in human islets promotes beta-cell function in terms of GSIS and insulin content. These accentuated effects also apply to EndoC-βH1 cells after 2-day culture but not to cells with rhCLEC11A administrated acutely. Moreover, the proliferation rate of beta-cells in 2-day and 7-day cultured human islets was dose dependently increased following exposure to different concentrations of rhCLEC11A. In EndoC-βH1 cells, rhCLEC11A accentuated the cell proliferation from the concentration of 10 ng/mL. Taken together, we assume that rhCLEC11A plays a role in triggering beta-cell insulin secretion and proliferation, probably via regulating intracellular signalling pathways. One recent study carried out in mouse focusing on osteogenesis has identified that ITGA11 is an osteolectin receptor, and the Wnt pathway could be activated by the binding of osteolectin to the ITGA11 (Shen *et al.* 2019). The expression of ITGA11 was confirmed in both human islets and EndoC-βH1 cells in this study. Furthermore, in beta-cells, the activation of Wnt signalling not only is involved in pancreas development and proliferation (Rulifson *et al.* 2007, Liu & Habener 2010) but also plays a role in regulating cell function of mature beta-cells (Fujino *et al.* 2003, Bader *et al.* 2016). We thus assume that CLEC11A promotes insulin secretion and proliferation in the human beta-cell, and it could probably be due to its binding to the recently discovered receptor ITGA11, followed by the regulation of intracellular signalling pathways such as activation of the Wnt signalling pathway.

In the later stages of type 2 diabetes, loss of transcription factors such as PDX1 and beta-cell-specific transcriptional activator MAFA was reported (Hunter & Stein 2017), and some studies have linked these transcription factors to the insulin biosynthesis and secretion in murine islets and beta-cells (Hagman *et al.* 2005, Zhu *et al.* 2017). Increased mRNA expression of *MAFA* and *PDX1* in rhCLEC11A-treated human islets and EndoC-βH1 cells was identified by RT-qPCR, which may contribute to the stimulatory effects of CLEC11A in beta-cell function in human islets and EndoC-βH1 cells. On the other hand, repeated exposure of beta-cells to high levels of fatty acids dampens beta-cell function in terms of GSIS and insulin content (Sako & Grill 1990, Cen *et al.* 2018), downregulates insulin gene expression (Jacqueminet *et al.* 2000), and reduces the expression of *Mafa* in rat islets (Hagman *et al.* 2005). These findings were also identified in EndoC-βH1 cells under long-term exposure to palmitate in this study. However, a very weak protective effect against palmitate-induced beta-cell dysfunction was identified, e.g. minor improvement in insulin stimulation index and increased *INS* mRNA expression level but no effect on the insulin content. Therefore, CLEC11A may play a regulatory role in palmitate-treated beta-cells but could not overcome the detrimental effects caused by chronic palmitate exposure. In contrast, our previous study carrying out RNAseq analysis in mouse pancreas found that obesity reduced the expression level of *Clec11a* and that the rhCLEC11A plays a regulatory role in proliferation and lipid metabolism in mouse islets (Shi *et al.* 2019). These discrepancies could be due to the differences in species, feeding behaviours, and diet compositions between rodents and humans, which may impact the islet and beta-cell physiology (Rorsman & Ashcroft 2018).

In conclusion, we have identified that CLEC11A is expressed in insulin-secreting beta-cells and glucagon-secreting alpha-cells in human islets. The findings that rhCLEC11A caused an increase in insulin secretion, insulin content, and proliferation are partially due to the induced mRNA expression levels of transcription factors *MAFA* and *PDX1* in human islets and EndoC-βH1 cells. CLEC11A, therefore, may provide a novel therapeutic target for maintaining beta-cell function in patients with diabetes.

## Supplementary Materials

Supplementary Material

## Declaration of interest

The authors declare no conflicts of interest.

## Funding

The study was generously supported by the National Natural Science Foundation of China (no. 82100845) (to RS) and the Swedish Juvenile Diabetes Foundation and the Ernfors family foundation (to JL).
