# Supplementary material for: CLEC11A improves insulin secretion and promotes cell proliferation in human beta-cells
Source: J Mol Endocrinol. 2023 Jun 21;71(1):e220066. doi: 10.1530/JME-22-0066 (PMC10326638; doi:10.1530/JME-22-0066)
Supplement: Supplementary Material [file supplementary_material.pdf]

**Supplementary files**

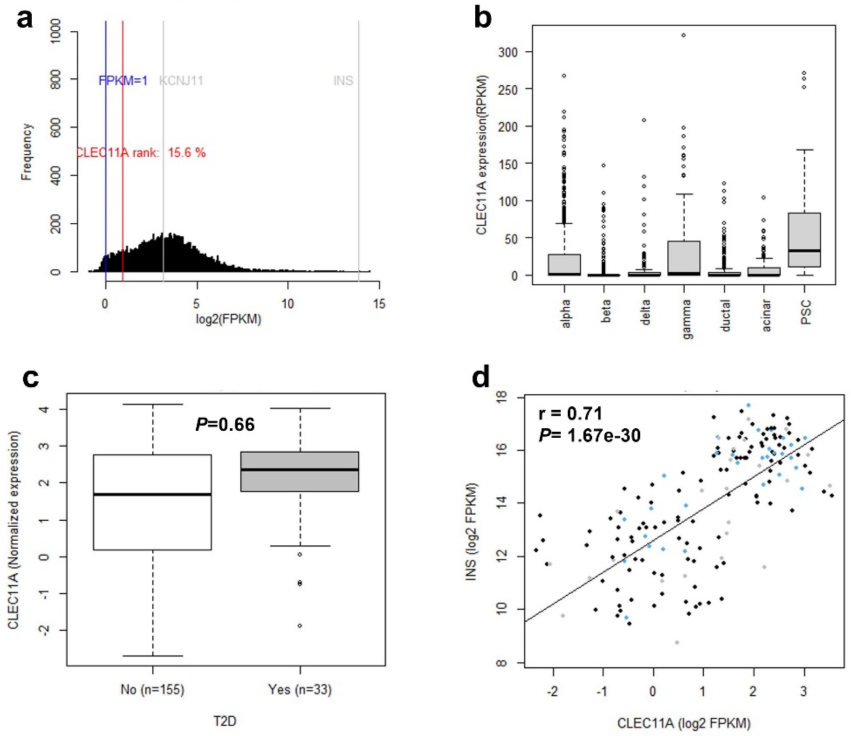


**Supplementary figure 1. *CLEC11A* gene expression in human islets generated from Islet Gene View (IGW) platform https://mae.crc.med.lu.se/IsletGeneView/ which applied RNA sequencing and genome-wide genotyping in human islets from 188 donors.** (a) Histogram of gene FPKMs. The expression of *CLEC11A* in relation to other genes in human islets. (b) *CLEC11A* gene in human islet cell-type expression. (c) *CLEC11A* gene expression in relation to T2D diagnosis. The statistical difference of normalized expression of *CLEC11A* gene in human islets between non-T2D and T2D: *P*=0.66. T2D, type 2 diabetes. (d) *CLEC11A* gene expression in human islets in relation to the insulin gene (*INS*) expression. Spearman's ρ (r) and the *P*-value of the gene based on the empirical correlation distribution are reported.

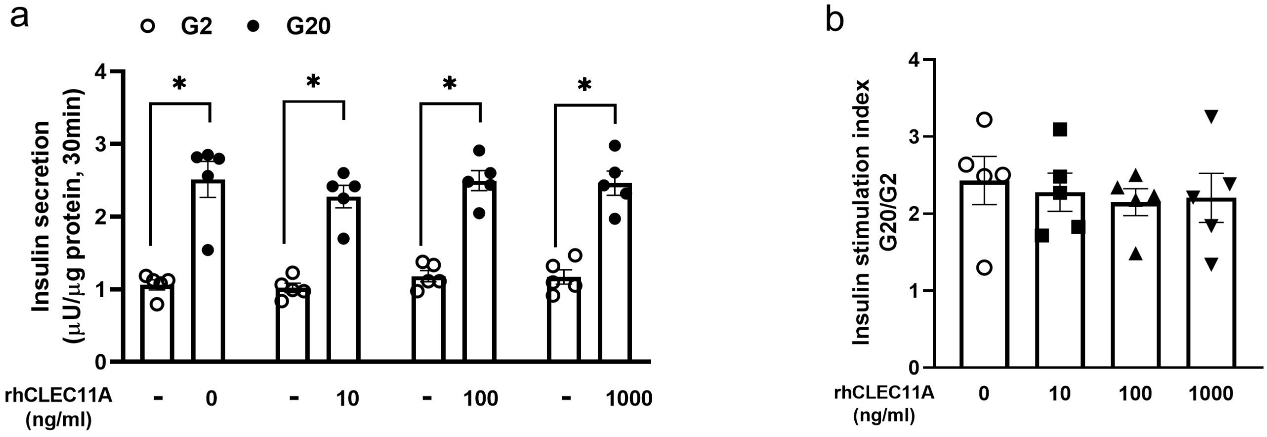


**Supplementary figure 2. Acute effects of rhCLEC11A on static GSIS in EndoC-βH1 cells.** (a) Static GSIS was performed on EndoC-βH1 cells with 2 mM glucose (G2) and 20 mM glucose (G20) for 30 min and rhCLEC11A with different concentrations (0, 10, 100, and 1000 g/ml, respectively) indicated was added during the 20 mM glucose exposure. Supernatant was collected and insulin secretion was measured. (b) Insulin stimulation index was calculated from static GSIS (a) by dividing the insulin secreted from cells stimulated by the combination of 20 mM glucose and different concentrations of rhCLEC11A (0, 10, 100, and 1000 ng/ml, respectively) by the insulin secreted from cells exposed to 2 mM glucose. Results are expressed as means ± SEM of n=5 independent experiments. * indicates $P<0.05$.

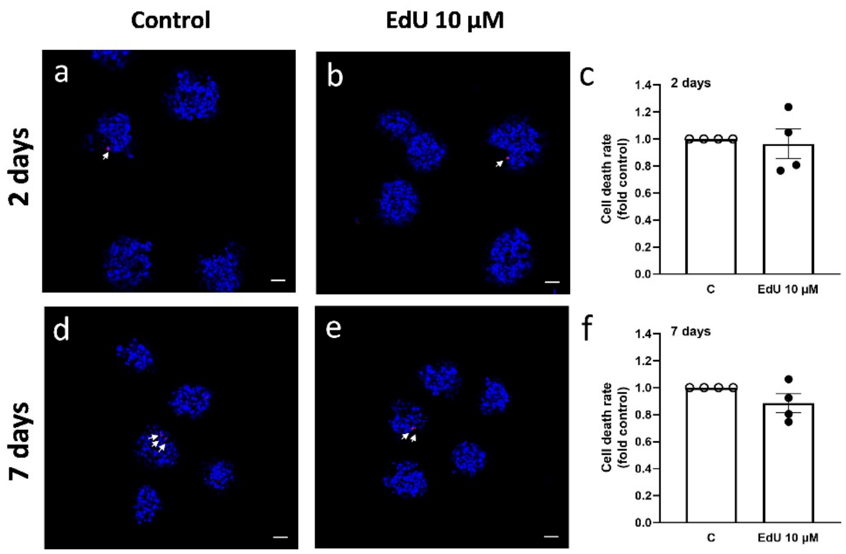


**Supplementary figure 3. Cell death in EdU-treated human islets.** Human islets (5-10 islets/group) were cultured with or without 10 µM EdU for 2 days and 7 days, respectively, followed by incubating with Propidium Iodide (ThermoFisher) (0.1 mg/ml) and Hoechst 33342 (ThermoFisher) (10 µg/ml) for 30 min in islet culture medium. Images were acquired by confocal microscopy (Zeiss LSM780) and the entire human islets were examined using z-stack program in the confocal microscopy. Images were analyzed using Image J software. (a, b, d, e) representative images with dead cells labelled by Propidium Iodide (red) and indicated with arrows (white), and nuclei (blue) was labelled by Hoechst 33342. The proportion of Propidium Iodide-staining cells in islet cells was calculated and expressed as fold control, as shown in (c) 2-day culture and in (f) 7-day culture. Scale bars, 50 µm. Results are expressed as means ± SEM of n=4 donors. * indicates $P<0.05$.

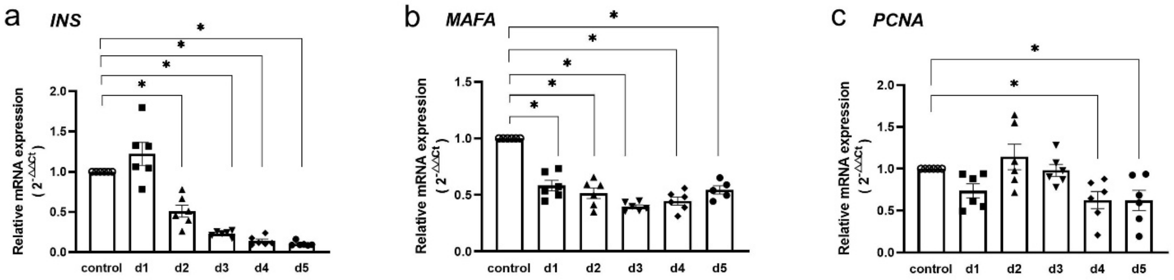


**Supplementary figure 4. Time course of palmitate effects in EndoC-βH1 cells.** EndoC-βH1 cells were cultured in the absence (control) and presence of 1.5 mM palmitate for different time periods. After culture, the relative mRNA expression levels of (a) *INS,* (b) *MAFA* and (c) *PCNA* were measured by RT-qPCR, normalized to endogenous control *GAPDH* and calculated with the $2^{-\triangle\triangle Ct}$ method, respectively. Results are means ± SEM of n=4–6 independent experiments. * indicates $P<0.05$

**Supplementary table 1. Donor information of human islets used in this study.**

| Donor Number | Age | Gender | BMI ($kg/m^2$) | HbA1c (mmol/mol) |
|---|---|---|---|---|
| 1 | 69 | M | 21.5 | 36 |
| 2 | 60 | M | 26.3 | 41 |
| 3 | 66 | M | 26.9 | 38 |
| 4 | 65 | M | 23.6 | 42 |
| 5 | 73 | F | 21.7 | 35 |
| 6 | 49 | M | 30.9 | 39 |
| 7 | 68 | F | 26.3 | 40 |
| 8 | 60 | M | 27.5 | 34 |
| 9 | 53 | F | 26.6 | 34 |
| 10 | 61 | M | 24.5 | 35 |
| 11 | 62 | F | 29.4 | 38 |
| 12 | 67 | F | 29.0 | 33 |
| 13 | 54 | F | 18.0 | 36 |
| 14 | 58 | F | 22.5 | 36 |
| 15 | 42 | M | 30.0 | 39 |

**Supplementary table 2. Primers for RT-qPCR experiments.**

| **Gene name** | **Forward primer** | **Reverse primer** |
|---|---|---|
| *CLEC11A* | 5’-ACATCGTCACTTACATCCTGGG-3’ | 5’-GTGTCCAACGCGTGCAG-3’; |
| *ITGA11* | 5’-AATAAGTGGCTGGTCGTGGG-3’ | 5’-CTGTTGTCCTTGGGGTTGGT-3’ |
| *PCNA* | 5’-CATGGGCGTGAACCTCACCAG-3’ | 5’-CGGCATATACGTGCAAATTCACC-3’ |
| *INS* | 5’-CAACACCTGTGCGGCTCACA-3’ | 5’-AAGAAGCCTCGTTCCCCGCA-3’ |
| *PDX1* | 5’-CCTTGTGCTCGGGTTATGTT-3’ | 5’-ATCATCCCACTGCCAGAAAG-3’ |
| *MAFA* | 5’-GCGGAGAACGGTGATTTCTA-3’ | 5’-AGGAAAGGAGGCTGAGAAG-3’ |
| *GAPDH* | 5’-GAGTCAACGGATTTGGTCGT-3’ | 5’-GACAAGCTTCCCGTTCTCAG-3’ |
| *ACTB* | 5’-CACCATTGGCAATGAGCGGTTC-3’ | AGGTCTTTGCGGATGTCCACGT-3’. |
